# Microfluidic Platforms for Modeling Cancer-Associated Thrombosis: Current Status and Future Directions

**DOI:** 10.1016/j.rpth.2026.106835

**Published:** 2026-07-13

**Authors:** Dongyue Fan, Henri H. Versteeg, Araci M.R. Rondon

**Affiliations:** Division of Thrombosis and Hemostasis, Department of Internal Medicine, Einthoven Laboratory for Vascular and Regenerative Medicine, Leiden University Medical Center, Netherlands

**Keywords:** cancer-associated thrombosis, hypercoagulability, microfluidics, models, neoplasms, thrombosis

## Abstract

Cancer-associated thrombosis (CAT) is the second leading cause of death among cancer patients. Its pathogenesis is multifactorial and involves dynamic interactions among tumor cells, platelets, coagulation factors, endothelial cells, immune cells, and blood flow. Traditional *in vitro* 2-dimensional models fail to accurately mimic the dynamic microenvironment of CAT in humans. Although animal models can mimic complex pathological conditions, significant differences in the composition and regulation of the coagulation system between animals and humans limit their ability to accurately model CAT in humans. In this review, we briefly outline the mechanisms involved in CAT to provide the biological rationale for the design of emerging microfluidic platforms. We then discuss how these platforms have advanced CAT research. The ability of microfluidic platforms to precisely control perfusion, cellular composition, and flow rate enables researchers to accurately simulate the human cancer-vascular microenvironment in vitro and investigate cancer-specific mechanisms of thrombosis. We further discuss the challenges that need to be addressed for microfluidic models to support basic and clinical research.

## Essentials

• Cancer-associated thrombosis (CAT) is a major cause of death in cancer patients.
• This review examines microfluidic models that mimic cancer and blood vessels.
• These systems recreate blood flow and cell interactions driving clot formation.
• They may improve drug testing and clot risk prediction but need standardization.

## Graphical abstract

**Figure:**
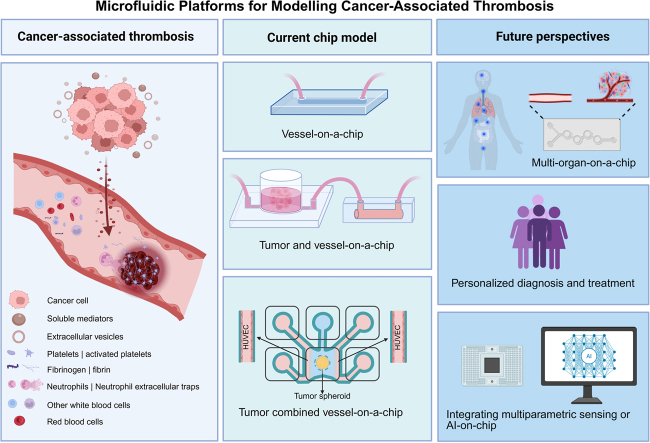

## 1 Introduction

Thrombosis is a common complication of cancer and the second leading cause of death in cancer patients, after the cancer itself [1]. Thrombosis occurs in up to 8% of all cancer patients, and conversely, ∼ 20% to 30% of all first venous thromboembolic events are cancer associated [2]. Compared with cancer patients without thrombosis, those who develop thrombosis have a 3.4-fold higher risk of death [3]. CAT can manifest as venous thrombosis, arterial thrombosis, and chronic disseminated intravascular coagulation [3], with venous thromboembolism (VTE) being the most frequent [4]. The risk of CAT is influenced not only by tumor-related factors, such as tumor site and stage, and treatment-related factors, including chemotherapy or surgery, but also by patient-related characteristics. These may include advanced age, obesity, immobility [5], a history of VTE, and inherited thrombophilia [5,6], such as factor (F)V Leiden and the prothrombin G20210A mutation, which have been associated with an increased risk of VTE in patients with cancer [7,8]. These factors highlight the complexity of CAT pathogenesis and underscore that the mechanisms underlying CAT remain highly complex and not yet fully understood [9].

The mechanisms underlying thrombosis in patients with cancer vary according to tumor type, further highlighting the biological complexity of CAT and the corresponding challenges in developing representative experimental models (Table 1) [[10], [11], [12], [13], [14], [15], [16], [17], [18], [19], [20], [21], [22], [23], [24], [25], [26], [27], [28], [29], [30], [31], [32], [33], [34], [35], [36]]. One important mechanism is the release of extracellular vesicles (EVs) by tumor cells [37] (Table 1). The procoagulant effect of these EVs is mainly achieved by providing negatively charged phospholipids, such as phosphatidylserine (PS), which serve as surfaces for the assembly of coagulation factor complexes. In addition, tumor cells can release EVs carrying procoagulant factors. For instance, EVs secreted by pancreatic cancer cells can promote coagulation through the exposure of PS/phosphatidylethanolamine and tissue factor (TF) on their surface [38]. TF-positive EVs released by tumor cells into the bloodstream, where they bind FVII/FVIIa, initiate the extrinsic coagulation pathway to generate FXa and thrombin, thereby promoting VTE [39,40]. In prostatic tumors, the release of EVs containing polyphosphates may activate the contact pathway [41]. In gliomas, microvesicles with podoplanin can promote coagulation by activating platelets [19].

**Table 1:** Potential mechanisms underlying CAT

Cancer type	Model	WBC/platelet changes	EVs	NETs	Soluble mediators	Contribution to thrombosis
Pancreatic cancer	Cell line; animal; clinical	Blood neutrophil counts ↑ [10]	TF+ EV ↑ [11] PSGL-1+ EVs ↑ [12] sEVs (ITGB2) ↑ [13]	citH3 (NETs biomarker) ↑ [14] Cell-free DNA ↑ [10]	Fibrinogen, FVIII, VWF, VEGF, PF4, P-Selectin, PAI-1↑ [15,16] IL-1, TNF-α ↑ [17] Antithrombin, protein C ↓ [17] CXCL13↑ [13]	Increased thrombin generation level Increased platelet activation Activated intrinsic contact pathway Impaired fibrinolysis system Increased inflammatory activity
Brain tumor	Clinical	WBC count ↑; Platelet count ↓ [18]	PDPN+ EVs ↑ [19]	—	—	Increased platelet activation
Lung cancer	Animal; clinical	WBC count ↑ [9] Platelet T cell aggregates ↑ [20] Circulating platelet count ↑ [21]	TF+ EVs ↑ [12] PDPN+ MP ↑ [22]	citH3 (NETs biomarker) ↑ [14]	ADAMTS-13 ↑ [16]	Increased thrombin generation Increased platelet activation
Colorectal cancer	Clinical	WBC count ↑ [9] Platelet and cancer cell interactions ↑ [23]	TF+ EVs (from platelets) ↑ [24]	—	VWF levels, VWF/ADAMTS-13 ratio ↑ [16] PAI-1, TAFI ↑ [25]	Increased platelet activation Impaired fibrinolysis system
Gastric cancer	Animal; clinical	Platelet count ↑ [9]	—	NETs formation ↑ [26]	VWF levels, VWF/ADAMTS-13 ratio ↑ [16]	Increased platelet activation
Esophageal cancer	Clinical	—	—	—	VWF levels, VWF/ADAMTS-13 ratio ↑ [16]	Increased platelet activation
Breast cancer	Animal; clinical	Platelet count ↑ [9]	TF+ EVs ↑ [27] sEVs (ITGB2)↑ ↑ [13]	G-CSF↑ [28] citH3 (the NETs biomarker) ↑ [14]	PAI-1 ↑ [29] CXCL13↑ [13]	Increased platelet activation Increased thrombin generation Impaired fibrinolysis system
Melanoma	Cell line; animal	—	EVs ↑ [30] sEVs (ITGB2)↑ [13]	—	CXCL13↑ [13]	Increased platelet activation Increased fibrin formation and thrombin generation
Ovarian cancer	Cell line	Platelet count ↑ [9]	—	—	IL-6 ↑ [31]	IL-6 associated with an increased incidence of VTE
Lymphoma	Cell line	—	—	—	FVIII ↑ [32]	Increased procoagulant activity
Bladder cancer	Cell line	—	Procoagulant EVs ↑ [33]	—	FVIII ↑ [32]	Increased procoagulant activity
Teratocarcinoma	Cell line	—	Procoagulant EVs ↑ [33]	—	—	Increased procoagulant activity
Hepatocellular carcinoma	Clinical	—	—	DNA–histone complex, double-stranded DNA, neutrophil elastase ↑ [34]	VWF ↑ [35] Protein C, protein S, and antithrombin ↓ [36]	Contact system activation Increased procoagulant activity Impaired fibrinolysis system

ADAMTS-13, a disintegrin and metalloproteinase with thrombospondin type 1 motif member 13; CAT, cancer-associated thrombosis; citH3, citrullinated histone H3; CXCL13, C-X-C motif chemokine ligand 13; EV, extracellular vesicle; F, factor; G-CSF, granulocyte colony-stimulating factor; IL, interleukin; NET, neutrophil extracellular trap; PAI, plasminogen activator inhibitor; PDPN, podoplanin; PSGL, P-selectin glycoprotein ligand; TF, tissue factor; TNF, tumor necrosis factor; VTE, venous thromboembolism; VWF, von Willebrand factor; WBC, white blood cell.

Tumor cells themselves can also promote CAT by inducing platelet activation [42] (Table 1). Tumor cells can release ADP [43], which binds to platelet receptors P2Y1 and P2Y12 to induce platelet degranulation, which is a key step in platelet activation [44]. In addition, elevated plasma levels of von Willebrand factor (VWF) and P-selectin in cancer patients have been associated with an increased risk of VTE. Elevated plasma VWF in cancer patients promotes platelet recruitment and aggregation and serves as a protective carrier for FVIII, thereby contributing to thrombus formation in malignancy [45]. Similarly, P-selectin, derived primarily from activated platelets and endothelial cells, promotes cellular adhesion and further contributes to thrombus formation [46]. Consistent with enhanced platelet activation, elevated levels of platelet microparticles have been observed in patients with solid tumors [47]. Moreover, an *in vitro* hemostatic model demonstrated that platelet-derived microvesicles are 50 to 100 times more effective in procoagulant activity (PCA) than thrombin-activated platelets [48]. In addition to these direct procoagulant effects, activated platelets can also act as inducers of NETosis, thereby further promoting CAT [49,50].

NETosis, the process of neutrophil extracellular trap (NET) formation has gained increasing attention in CAT (Table 1). NETs are large extracellular web-like structures released by activated neutrophils that can capture, neutralize, and kill pathogens [51]. They are composed of DNA, histones, and various granule proteins [52]. However, NETs have also been recognized as a key player in thrombosis. Free DNA within NETs can promote the formation of FXIIa through the contact pathway, thereby enhancing coagulation [53]. Histones can activate platelets via Toll-like receptors 2 and 4 [54]. In addition, activated platelets bind to P-selectin glycoprotein ligand-1 on the surface of neutrophils through platelet surface expressed P-selectin, a process that enhances neutrophil activation and promotes NET formation [55]. Tumor-released interleukin 8 and granulocyte-colony stimulating factor can promote the formation of NETs [28], thereby activating neutrophils and inducing NET release, ultimately promoting CAT.

In addition to activating procoagulant pathways, tumors may also promote thrombosis by suppressing fibrinolysis process and anticoagulant system. Fibrinolysis is the physiological process responsible for fibrin degradation and thrombus resolution. Under normal physiological conditions, fibrinolysis is initiated when the plasminogen activators such as tissue-type plasminogen activator and urokinase-type plasminogen activator convert plasminogen into plasmin, which subsequently degrades fibrin and dissolves the thrombus [56]. Plasminogen activator inhibitor 1, which suppresses the fibrinolytic system, promotes fibrin deposition and leads to fibrin thrombus formation [57]. Studies have shown that increased plasminogen activator inhibitor 1 activity is independently associated with a higher risk of VTE in patients with pancreatic and breast cancers [15,29]. Furthermore, reduced activity of natural anticoagulant pathways also contributes to CAT. Under physiological conditions, thrombin bound to endothelial thrombomodulin (TM) activates protein C (PC) to form activated PC (APC), in concert with protein S, inactivates FVa and FVIIIa, thereby limiting further thrombin generation [58]. In cancer, endothelial damage and dysfunction caused by tumor-associated inflammation or chemotherapy may lead to downregulation of TM, dysfunction of the PC pathway, and acquired APC resistance, thereby enhancing thrombin production even in the absence of hereditary FV Leiden [57,59]. Lower plasma levels of antithrombin and PC were observed in patients with pancreatic cancer and hepatocellular carcinoma than those in controls [17,36]. Notably, in patients with gastrointestinal, breast, lung, and genitourinary cancers, impaired PC pathway function was independently associated with VTE risk [59]. In contrast to the reduced anticoagulant activity observed in the PC pathway, the association between TF pathway inhibitor (TFPI) and CAT appears more complex. TFPI is a physiological inhibitor of the TF–FVIIa pathway, and elevated circulating TFPI levels have been associated with an increased risk of VTE [60]. This suggests that increased TFPI may reflect a compensatory response to cancer-associated hypercoagulability. Although cancer is clearly associated with coagulation abnormalities, CAT is mechanistically complex and cannot be explained by a single unified pathway. Multiple molecular and cellular mechanisms have been implicated, including TF-driven coagulation, platelet and neutrophil activation, endothelial dysfunction, impaired fibrinolysis, and disruption of natural anticoagulant pathways. However, their relative contributions and interactions likely vary across tumor types, stages, and treatment and, therefore, remain incompletely understood.

Given the multifactorial nature of CAT and the absence of a unifying mechanistic framework, various experimental and clinical models have been established to investigate CAT. Conventional research models include 2-dimensional (2D) cell cultures, animal models, and patient-based clinical studies. While traditional 2D culture models based on cell monolayers have been widely used in the study of CAT, these models cannot accurately mimic the complex *in vivo* interactions within the tumor microenvironment that presumably play a role in CAT [61]. For example, these models lack physiological shear stress generated by blood flow and fail to reproduce the complex interactions among tumor cells, endothelial cells, immune cells, and blood components in circulation [62]. Another widely used model for studying CAT is the mouse model, which provides a complete physiological system for investigating CAT [63]. These models have been widely used to investigate thrombus formation in different types of cancer, such as lung, breast, and pancreatic cancers [12,27,64]. However, establishing mouse models of CAT often requires the use of immunodeficient mice transplanted with human tumors. While this approach allows direct investigation of the effects of human tumors on thrombosis, it neglects the crucial role of the immune system in thrombus formation [65]. Alternatively, immunocompetent mice bearing murine tumors (syngeneic models) may be used, but such models cannot directly recapitulate the characteristics of human tumors or fully represent their influence on thrombosis [66]. In addition, mice possess a more active anticoagulant system, such as higher activity of the anticoagulant mediators APC [67]. Therefore, when using mouse thrombosis models, thrombosis generally needs to be artificially induced by surgically inducing endothelial injury, hypercoagulability, or stasis, making these models highly invasive and prone to artifacts, thus poorly representing the spontaneous thrombus formation observed in human cancer patients [68]. Clinical studies remain the gold standard for establishing the association between cancer and thrombosis [69]. However, they are inherently observational and, therefore, cannot provide mechanistic insights. Although cancer significantly increases the risk of VTE, the total number of cancer-associated VTE events is still, at up to 8% of all cancer cases, and most clinical studies, therefore, rely on mixed cohorts that include multiple tumor types [70]. Patient heterogeneity, such as differences in tumor type, tumor stage, and treatment, further obscures our understanding of CAT. In addition, the inability to achieve real-time visualization of intravascular coagulation processes in patients limits the investigation of the dynamic microenvironment underlying thrombus formation.

In recent years, advances in organ-on-a-chip technology, combining 3-dimensional (3D) cell culture with microfluidics, have made it possible to replicate the microphysiological tumor environment in combination with thrombus formation in cancer patients [71]. Organ-on-a-chip devices offer excellent adaptability for a number of reasons. First, they can be designed to mimic the structure of human blood vessels and to reproduce physiological dynamic conditions by precisely controlling flow rates [72]. In addition, by incorporating multiple cellular components, they enable the interaction among tumor cells, endothelial cells, blood components, and immune cells [73]. By establishing high-throughput testing platforms, research efficiency can be significantly improved. Microfluidic platforms for studying CAT can recapitulate patient-specific *in vivo* characteristics, providing new insights into the mechanisms of thrombosis, drug screening, individualized risk prediction, and therapeutic strategies [62]. In this review, we briefly summarize the mechanisms of CAT that are relevant to the design of microfluidic and organ-on-a-chip models and discuss how these technologies have advanced the study of CAT. We focus on the application of microfluidic platforms in the study of CAT, including the design, fabrication, and characterization of microfluidic chips for modeling CAT, as well as their application in exploring the underlying mechanisms of CAT. Finally, we discuss the potential of microfluidic systems in drug screening and the development of new therapeutic approaches. The aim is to provide a reference for researchers in the field and to offer insights for the design of more advanced CAT-on-a-chip models.

This article was conducted as a narrative review rather than a systematic review. To improve the transparency of article selection, a structured literature search was performed on November 6, 2025 in Web of Science and PubMed. Search terms included combinations of CAT, cancer, organ-on-a-chip, and microfluidic, vascular, or endothelialized chip models. No restrictions on publication year or language were applied. Articles were included if they addressed CAT using organ-on-a-chip or microfluidic platforms, including models of thrombus formation, platelet or coagulation activation under flow, endothelialized vascular systems, CAT-related risk assessment, or drug screening. Articles mainly focused on how thrombotic components promote tumor invasion, metastasis, or tumor progression were excluded, unless they directly addressed CAT formation, thrombosis modeling, or VTE risk assessment. Because this was a narrative review, no formal risk-of-bias assessment or meta-analysis was performed.

## 2 Fabrication of Cat-On-A-Chip Models

Currently, microfluidic chips used to study CAT can be broadly categorized into 3 types: (1) single-chip simplified vascular-like channel systems models systems that directly use whole blood or plasma from cancer patients as the perfusate; (2) serial connection between tumor and vascular microfluidic modules in CAT modeling in which tumor cells or their conditioned media are introduced into the microfluidic channels; and (3) integrated chip systems that combine a tumor culture module with a vascular module.

The fabrication of organ-on-a-chips for CAT models primarily relies on established techniques such as soft lithography, which is favored due to its cost-effectiveness, high resolution, and design flexibility. Commercial platforms (eg, OrganoPlate, μ-Slide, and Vena8) further improve reproducibility and accessibility (Table 2) [[84], [77], [81], [82], [74], [75], [76], [78], [79], [80], [83]]. In addition to the design and fabrication of microfluidic devices, the biological configuration of the model, including cell composition, extracellular matrix, and flow conditions, is equally important in determining physiological relevance [73] (Table 2).

**Table 2:** Microfluidic platforms for modeling CAT

Research focus	Cancer type	Fabrication	Flow system	Perfusate	Matrix	Cell components	Mechanistic focus	Read out
Platelet activation predicts thrombotic risk in various cancers [4]	Breast cancer, liver cancer	Soft lithography in PDMS Channel diameter, 150 μm	Syringe pump; flow at *γ* = 1000/s (representative arterial shear rate)	Modified Tyrode buffer	Fibrin network formed by thrombin and fibrinogen	Labeled platelets from lung, liver, and breast cancer patients and healthy donors with CellMask Orange	Platelet activation	Platelet adhesion
sEVs derived from interstitial lung macrophages are key contributors to the increased risk of thrombosis [13]	Pancreatic ductal adenocarcinoma, breast cancer, melanoma, leiomyosarcoma, ovarian cancer, thymic lymphoma, and colon cancer	μ-Slide IV0.4 (Ibidi) Channel 17 × 3.8 × 0.4 mm (L × W × H)	Syringe pump; flow at 0.05 dyn/cm (representative venous shear rate)	Platelet-rich plasma from C57BL/6J mice	—	Labeled platelets with DyLight649-conjugated anti-GPIbβ antibody; sEV	Tumor-derived CXCL13 reprograms lung IMs to drive integrin β2^+^ sEV-mediated platelet aggregation, leading to thrombosis	Platelet adhesion
REG4 shows no impact on hemostasis in colorectal cancer [74]	Colorectal cancer	OrganoPlate 2-lane (Mimetas BV); glass, proprietary polymers; channel, 400 × 220 μm (W × H)	Gravity driven; flow at 0-0.8 dyne/cm^2^ (representative low venous shear rate)	EGM2 or RKO-REG4 expressing cell supernatants. Normal pool plasma	Collagen I	HUVECs	REG4 shows no direct effect on coagulation, thrombin generation, or endothelial activation in CAT models	Thrombin generation
Effects of tumor cells and chemotherapy on platelet activation rely on endothelial cells [75]	Breast cancer	Soft lithography in PDMS; channel diameter, 1 mm	Syringe pump; flow at 30 μL/min (representative venous shear rate)	Modified Tyrode buffer; 0 to 4 μM doxorubicin	Collagen II	MCF7 breast cancer cells; HUVECs; THP1 cells; platelets from healthy donors were labeled with an orange fluorescent dye	Endothelial injury induced by chemotherapy or tumors mediates platelet adhesion and activation	Platelet adhesion
Doxorubicin induces endothelial injury, thereby enhancing the procoagulant activity of tumor-derived MPs [76]	Ovarian clear cell carcinoma, glioblastoma	μ-slide I Luer (Ibidi); channel dimensions (L × W × H), 50 × 5 × 0.4 mm; μ-Slide III 3D	Syringe pump flow at 4 μL/min (representative venous shear rate)	Tumor culture medium with or without doxorubicin	Gelatin type B	ES-2, U87; HUVECs	Endothelial injury induced by DOX or tumors promotes endothelial procoagulant activity	PCA
Glioblastoma spheroids induce hypercoagulability [77]	Glioblastoma	OrganoPlate Graft (Mimetas BV); glass, proprietary polymers; channel, 400 μm × 220 μm × 2.5 mm (W × H × L)	Gravity driven; flow at 0-0.8 dyne/cm^2^ (representative low venous shear rate)	EGM2 or human plasma; rivaroxaban	Collagen type I	U251 glioblastoma spheroids; HUVECs	Glioblastoma spheroids overexpressing TF enhance endothelial procoagulant activity, thereby promoting thrombin generation in normal pool plasma	Endothelial permeability; thrombin generation
JAK-STAT inhibition modulates endothelial prothrombotic activation and leukocyte adhesion in MPN [78]	MPN	Soft photolithography; PDMS; channel, 405 μm × 1000 μm × 50 μm (L × W × H)	Syringe pump; flow at 1 dyn/cm^2^ (representative venous shear rate)	Whole blood from healthy donors and JAK2V617F^+^ MPN patients; 10 ng/mL TNFα; ruxolitinib; fedratinib	Gelatin	HUVECs; whole blood samples were labeled with calcein live stain (BioLegend) for leukocyte and platelet labeling	JAK inhibitors reduce JAK-STAT–driven endothelial activation and leukocyte endothelial interactions	Leukocytes rolling velocity
Ibrutinib affects atherothrombus formation in hematologic malignancies [79]	Chronic lymphocytic leukemia, mantle-cell lymphoma, Waldenström macroglobulinemia	Polycarbonate block; a nonstenotic channel (50 μm × 3 mm × 30 mm); stenotic channel (50 μm × 0.5 mm × 30 mm)	Flow at 1500/s; 3900/s at the stenotic region (representative arterial shear rate)	Whole-blood from healthy donors and hematologic malignancy patients with or without ibrutinib treatment	Collagen type I; a thin homogeneous layer of human atherosclerotic plaque material	Whole blood samples were labeled with DiOC6 for platelets labeling	Btk-dependent platelet adhesion and activation drive thrombosis under high shear, which is reduced by ibrutinib	Platelet adhesion
Vascular tumor–associated thrombosis [80]	Vascular tumors	Soft lithography in PDMS; channel, 200 μm × 50 μm (W × H)	Syringe pump; flow at 0.5-1.0 μL/min (representative venous shear rate)	Whole blood from healthy donors	Fibronectin	Whole blood samples were supplemented with fluorescent beads to assess flow dynamics	Abnormal blood flow induces a prethrombotic state, promoting aggregation of RBCs with proteins and platelets	Multiaggregates of RBCs and proteins/platelets
Bladder cancer–induced endothelial activation and hypercoagulation [81]	Bladder cancer	IBIDI μ-slide; glass, optically clear polymer; channel, 5 mm × 0.2 mm × 50 mm (W × H × L)	Air pressure–based pump; flow at 6 dyn/cm^2^ (representative venous shear rate)	HRBS; tumor supernatant	Gelatin	Bladder cancer cell lines (RT4, T24, RT112, and UROtsa); HUVECs were stained with CellTracker Blue; erythrocytes; platelets were labeled with calcein red-orange	Tumor-derived VEGFA activates endothelium to release VWF, promoting platelet adhesion and thrombosis	Platelet aggregation
Tumor microvesicle adhesion to endothelium under flow [82]	Ovarian cancer and glioblastoma	μ-Slide I Luer (Ibidi); Vena8 endothelial cell biochip (Cellix); channel, 800 μm × 120 μm × 28 mm (W × D × L)	Syringe pump; flow at 4 μL/min (representative venous shear rate)	Tumor-conditioned medium containing CFSE or FITC-labeled microvesicles (ES-2 or U87 origin)	Gelatin type B	ES-2 and U87 cells were labeled with CFSE to generate fluorescent tumor-derived microvesicles; HUVECs	Tumor-derived microvesicles bind to endothelial cells under flow, promoting thrombosis	EV attachment to endothelial cells
Platelet strings on tumor-activated endothelium [83]	Melanoma and colon cancer	Piezoelectric crystal (LiNbO_3_); 10 mm	Surface acoustic waves drive (electrodes); (representative venous shear rate)	HEPES-buffered Ringer solution with nonactivated human platelets; tumor supernatant; FN-439 (MMP-1 inhibitor)	Gelatin	HT-29 and A7; HUVECs, platelet strings were visualized by immunofluorescence using anti-VWF and anti-CD41 antibodies with Qdot conjugated secondary antibodies	In melanoma and colon cancer, tumor-derived MMP-1 activates endothelial PAR-1, leading to VWF release and platelet adhesion	Platelet adhesion

CFSE, carboxyfluorescein succinimidyl; DOX, doxorubicin; EGM2, endothelial growth medium-2; FITC, fluorescein isothiocyanate; FN-439, matrix metalloproteinase-1 inhibitor; HRBS, HEPES-buffered Ringer’s solution; HUVEC, human umbilical vein endothelial cell; IM, interstitial macrophage; JAK, Janus kinase; JAK2V617F^+^ MPN patients, patients with JAK2 V617F-positive myeloproliferative neoplasms; MPN, myeloproliferative neoplasm; PCA, procoagulant activity; PDMS, polydimethylsiloxane; RBC, red blood cell; REG4, regenerating islet-derived protein 4; RKO-REG4, REG4-overexpressing RKO human colorectal cancer cells; sEV, small extracellular vesicle; STAT, signal transducer and activator of transcription pathway; TNF, tumor necrosis factor; VEGF, vascular endothelial growth factor.

CAT-on-a-chip systems typically incorporate endothelialized microchannels, most commonly using human umbilical vein endothelial cells (HUVECs), combined with extracellular matrix such as fibrin or collagen to support adhesion and mimic vascular microenvironment [[85], [86], [87], [88]]. Flow is usually controlled by syringe pumps to reproduce pathological shear stress conditions [89]. More advanced models integrate tumor cells, patient-derived blood samples, or immune cells (eg, THP-1, a monocyte cell line) to recapitulate tumor, vascular, and immune interactions [75] (Table 2). Detection strategies vary depending on the research, including platelet adhesion, thrombin generation assays, and formation of the clot-like multiaggregates of red blood cells and platelets, tumor cells, and tumor cell–derived EVs [77,82,80] (Table 2).

## 3 Insights into Cat Using Organ-On-A-Chip Models

Tumor-derived EVs are a major focus in studies of the mechanisms underlying CAT. Previous studies based on animal models and clinical cohorts have shown that EVs can promote CAT by activating platelets and triggering the extrinsic coagulation pathway. In recent years, the application of microfluidic systems to CAT research has enabled investigation of the effects of EVs on the vascular endothelium. Algarni et al. [76] used a dual microfluidic chip consisting of a tumor chamber and a microvascular channel to model the interaction between tumor-derived EVs and the vascular endothelium under physiologically flow conditions (Figure Ai). The study showed that tumor-derived procoagulant EVs, particularly TF-positive EVs, were able to bind to the surface of HUVECs under flow conditions. Meanwhile, when tumor conditioned medium rich in EVs was perfused through the endothelialized microvascular channel, the number of EVs in the medium was markedly reduced, and the PCA also decreased. These results indicate that the abundance of EVs in the culture medium is closely related to its procoagulant potential. However, in this study, the procoagulant effect of EVs was mainly based on indirect evidence of changes in EVs levels and PCA in the medium, rather than on direct measurement of procoagulant changes in the endothelial cells themselves. Kapteijn et al. [77] further used the OrganoPlate 2 lane chip to generate artificial microvessels and established a perfusion system mimicking venous flow using a rocker platform by adjusting the platform tilt angle and rocking interval. In this study, artificial microvessels were preincubated for 4 hours with EVs derived from TF-overexpressing U251 cells (Figure Bi). After removal of the EV-conditioned medium, normal pooled plasma (NPP) was perfused through the microvessels. Artificial vessels pretreated with EVs showed increased thrombin generation peak and endogenous thrombin potential (ETP), suggesting that exposure to EVs derived from TF overexpressing U251 cells enhanced the procoagulant potential of the endothelial microvascular environment. However, directly introducing tumor-conditioned medium into microvascular chips represents a relatively artificial and simplified approach. To better recapitulate the microenvironment of CAT *in vivo*, Kapteijn et al. [77] further developed a coculture model of glioblastoma U251 spheroids and artificial microvessels using the commercial OrganoPlate Graft platform. In this model, tumor spheroids were placed in the central graft compartment and connected to the artificial microvessels through the extracellular matrix (Figure Bii). To some extent, this model better mimics the interactions between tumors and blood vessels in the *in vivo* microenvironment. The results showed that tumor spheroids, particularly those overexpressing TF, increased endothelial permeability and significantly enhanced thrombin generation in NPP within the artificial vessels. Both a TF coagulant function–blocking antibody and rivaroxaban, an anticoagulant that inhibits FXa, the upstream activator of thrombin, markedly inhibited this procoagulant effect. These findings not only support a key role of TF in glioblastoma-associated hypercoagulability but also suggest that this platform may serve as a useful tool for investigating thrombosis mechanisms associated with other tumors and for screening anticoagulant strategies.

Endothelial dysfunction is a key contributor to thrombus formation. In CAT, endothelial injury caused by chemotherapeutic agents or tumor-derived factors has therefore become an important area of investigation [90,91]. Microfluidic platforms offer a physiologically relevant human cell–based system that integrates controlled perfusion, defined shear stress, and vascularized architecture. This enables direct and dynamic assessment of functional vascular and hemostatic end points, including endothelial permeability, blood cell adhesion, and thrombin generation. Accordingly, these systems are particularly valuable for elucidating how tumor- or chemotherapy-induced endothelial injury promotes CAT under flow conditions. To investigate the effects of tumors and chemotherapy on endothelial injury, Algarni et al. [76] developed a microfluidic platform in which 3D tumor spheroids were connected in series to a microvessel formed by HUVECs (Figure Ai). This study showed that microvessels treated with doxorubicin exhibited increased PCA, along with reduced endothelial cell viability. These results indicate that chemotherapy-induced endothelial injury may contribute to a more procoagulant endothelial surface phenotype. Similarly, Hao et al. [75] developed a microfluidic platform integrating a drug concentration gradient generator, a tumor cell culture chamber, and an endothelialized microvascular channel (Figure Aii). This system enabled the evaluation of endothelial and platelet injury, as well as platelet–endothelial cell adhesion, under stable flow across doxorubicin gradients. Their findings indicate that breast tumor cell–derived factors have cytotoxic effects on both endothelial cells and platelets and that doxorubicin-induced platelet activation largely depends on endothelial cells. These studies similarly highlight that thrombotic risk associated with tumors and chemotherapy is closely linked to endothelial injury under flow conditions and its impact on interactions between blood cells and the vascular wall.

Beyond endothelial injury induced by tumors or chemotherapy, tumor-associated structural vascular changes may also contribute to thrombosis in vascular tumors by disrupting local hemodynamics [92]. Llenas et al. [80] developed a serpentine vessel-on-a-chip platform to simulate the tortuous and irregular vascular geometry in vascular tumors (FigureC). This study showed that alterations in vascular geometry can lead to heterogeneous local hemodynamics, particularly in tortuous and constricted regions, where erythrocyte trajectories are altered and flow velocity is changed. Furthermore, under proadhesive conditions, perfused blood rapidly formed clot-like aggregates composed of erythrocytes, proteins, and platelets on the fibronectin-coated channel walls. These aggregates further induced local luminal narrowing, leading to alternating acceleration and deceleration of blood flow and thereby amplifying prothrombotic flow disturbances. This study highlights that CAT is not only a result of altered procoagulant phenotypes but also closely related to abnormal vascular structures and the abnormal blood flow.

**Figure:**
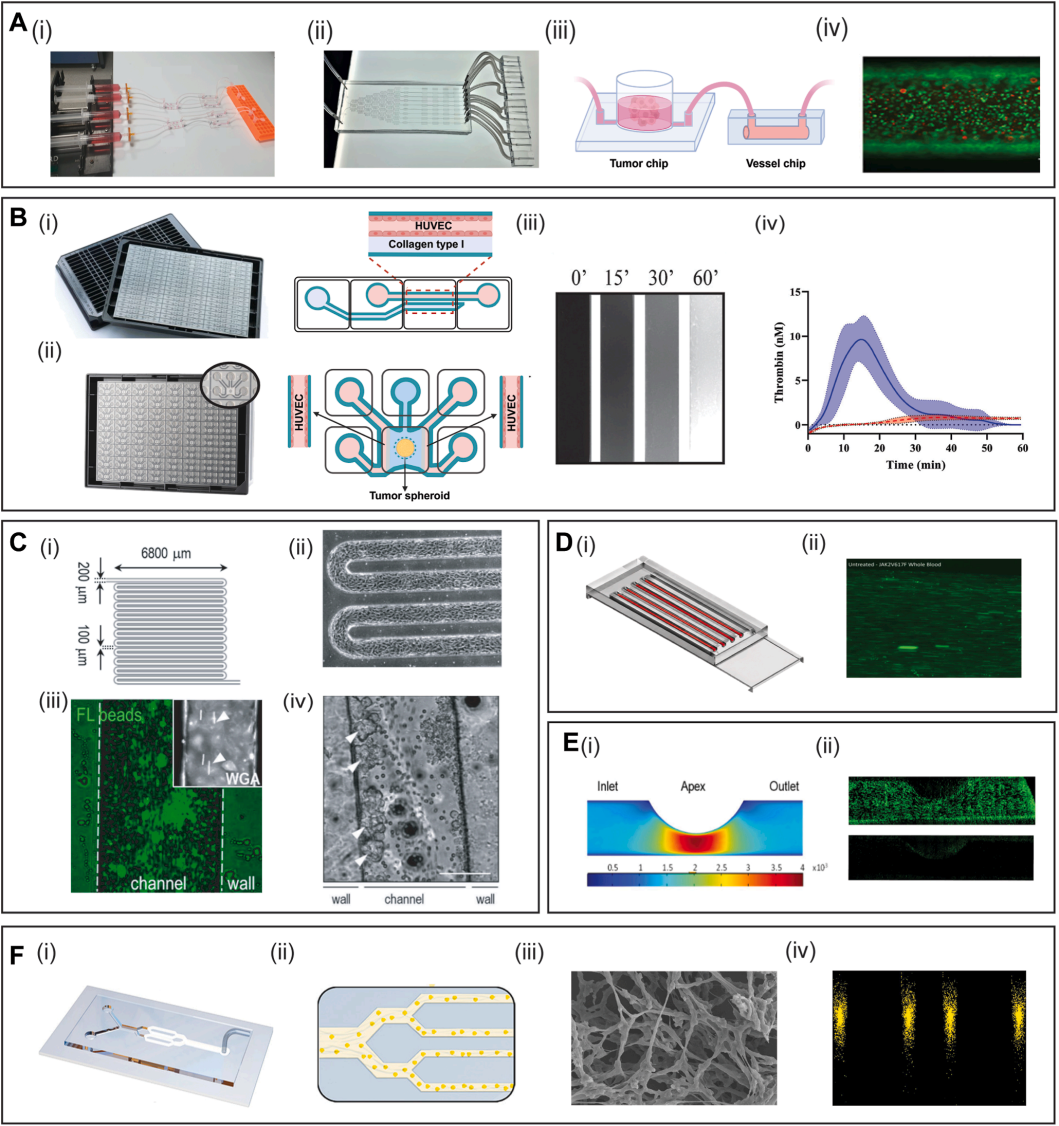
Representative models of CAT-on-a-chip. (A) Serial connection between tumor and vascular microfluidic modules in CAT modeling. (i) Microfluidic setup in which media is perfused through a μ-Slide III 3D chip containing a tumor spheroid and into downstream μ-Slide I Luer channels lined with human umbilical vein endothelial cells (HUVECs). *Source:* Adapted from Algarni et al. [76]. (ii) Microfluidic setup integrating a gradient generator, the tumor culture chips, and endothelialized microvascular channels in a sequentially connected configuration. *Source:* Adapted from Hao et al. [75]. (iii) Schematic diagram of serially connected microfluidic modules, consisting of a tumor culture chip followed by a microvascular chip. (iv) Fluorescence image showing platelet adhesion (orange) within the artificial vessel (green). *Source:* Adapted from Hao et al. [75]. (B) OrganoPlate platform for modeling CAT. (i) Formation of an artificial vessel in the OrganoPlate 2-lane configuration, used to investigate the effects of tumor-derived extracellular vesicles, supernatant, or patient plasma on thrombus formation. (ii) Coculture model established in the OrganoPlate Graft platform, enabling the integration of a tumor spheroid with the endothelialized vessel to mimic the tumor–vascular interface in CAT. (iii) Thrombin generation on-a-chip, with fluorescence reflecting real-time thrombin formation within the artificial vessel. (iv) Representative quantification of thrombin generation over time. *Source:* Adapted from Kapteijn et al. [77]. (C) A serpentine vessel-on-a-chip platform to mimic abnormal structure of vascular tumors. (i) Schematic diagram of a microvascular chip with a serpentine morphology. (ii) Serpentine morphology microvascular chip covered by HUVECs. (iii) The trajectories of fluorescent beads reflect the combined effects of red blood cells and endothelial cells on bead transport behavior under flow conditions. (iv) To mimic the procoagulant endothelial surface under pathological conditions, the endothelial layer was replaced with a clot-activating fibronectin coating on the channel walls, leading to the formation of clot-like red blood cell aggregates under the flow. *Source:* Adapted from Llenas et al. [80]. (D) Vessel on-a-chip perfused with blood from cancer patients. (i) Schematic diagram of a microvascular chip. (ii) Representative fluorescence images showing the flow of labeled leukocytes and platelets in whole blood samples. *Source:* Adapted from Beckman et al. [78]. (E) Shear-dependent thrombus formation on atherosclerotic plaque in a 60% stenotic chamber. (i) Computational shear-rate mapping. (ii) Representative fluorescence images of platelet accumulation (green) in the stenotic vessel-on-a-chip after perfusion of blood from patients not receiving (top) or receiving ibrutinib (bottom). *Source:* Adapted from Karel et al. [79]. (F) Fibrin networks based microfluidic model for assessing platelet activity. (i) Schematic layout of the microfluidic device showing the inlet and outlet configuration and microchannel geometry. (ii) Schematic illustration of fibrin network distribution within the microchannels. (iii) Scanning electron microscopy images of activated platelets adhering to the fibrin scaffold under microfluidic flow conditions. (iv) Representative fluorescence images showing platelet adhesion (yellow) to the fibrin network. Adapted from Li et al. [4].

Microfluidic platforms hold considerable promise for drug evaluation in CAT by enabling high throughput screening under physiologically relevant vascular conditions, including controlled flow, shear stress, defined vascular geometry, and endothelialized channels [93]. Thus, these platforms provide a physiologically relevant framework for assessing drug-induced prothrombotic and antithrombotic effects [94]. Beckman et al. [78] developed a microfluidic system with single channel to evaluate the potential prothrombotic effects of Janus kinase (JAK)1/2 inhibitors used for treating myeloproliferative neoplasms by measuring platelet and leukocyte flow velocities within microvessels [78] (FigureD). Their findings suggest that, contrary to previous assumptions that JAK-STAT inhibitors might increase the risk of thrombosis, JAK-STAT inhibitors such as ruxolitinib and fedratinib can reduce endothelial procoagulant and adhesive activation, thereby lowering the risk of thrombosis in JAK2V617F-positive myeloproliferative neoplasm [78]. Karel et al. [79] developed a microfluidic model that simulates atherothrombus formation under stenotic flow and incorporates perfusion of blood from patients with B-cell lymphoma to mimic the environment of CAT (FigureE). The study showed that the Bruton tyrosine kinase inhibitor ibrutinib, markedly reduced platelet adhesion and thrombus formation on plaque components but not on collagen in high shear stenotic regions, indicating a selective inhibitory effect on cancer-associated arterial thrombosis under high shear conditions.

Besides mechanistic studies and drug evaluation, another important application of microfluidic platforms in CAT concerns its promise as a functional tool for assessing VTE risk in cancer patients. Li et al. [4] constructed a fibrin network within microchannels to monitor platelet binding under physiologically relevant flow conditions, enabling assessment of tumor-associated platelet activation status (FigureF). The study showed that platelet activity increased with tumor progression and was associated with an increased risk of thrombosis. During the 6-month follow-up, the microfluidic platelet activity readout achieved an area under the curve (AUC) of 0.842 for predicting subsequent thrombotic events. The AUC reflects the overall ability of captured platelet abundance in the microfluidic chip to discriminate between advanced cancer patients who subsequently developed thrombotic events and those who did not during follow-up. An AUC of 0.842 indicates good discriminatory performance, performing better than traditional coagulation parameters such as thrombin time, activated partial thromboplastin time, prothrombin time, and fibrinogen. However, this model did not incorporate contributions of the endothelium, as it mainly relied on a fibrin network constructed in nonendothelialized microchannels to capture activated platelets. , In contrast, Fan et al. [95] relied on an endothelium-centered microfluidic strategy to assess thrombosis risk, more specifically with regard to endothelial procoagulant status induced by cancer patient plasma [95]. This study established HUVEC-based artificial vessels using the OrganoPlate 2 lane platform. Plasma from patients with gastrointestinal cancer was incubated with the artificial vessels for 4 hours, followed by removal of patient plasma and perfusion with NPP for thrombin generation assays. The results showed that plasma from patients with esophageal cancer who developed VTE during follow-up significantly enhanced peak and ETP compared with plasma from the non-VTE esophageal cancer group. Further analysis indicated that elevated peak and ETP were associated with a higher 6-month risk of VTE in patients with esophageal cancer, whereas a clinically developed and validated risk score based on the site of cancer, platelet count, hemoglobin and/or use of erythropoiesis-stimulating agents, leukocyte count, and body mass index—the Khorana score—showed no significant predictive value in this subgroup. This platform enabled evaluation of the endothelial procoagulant transformation induced by plasma from patients with cancer. These findings suggest that microfluidic functional models may complement traditional risk scoring systems and improve VTE risk stratification in specific cancer types by detecting patient plasma procoagulant state.

## 4 Current Limitations and Design Considerations

Despite promise, relative to tumor-on-a-chip and thrombosis-on-a-chip platforms, the development of CAT chips remains in an early developmental stage. This is primarily reflected in the reliance on immortalized tumor cell lines and HUVECs derived from the umbilical vein. In the real physiological environment, tumor tissues are composed of multiple cell types, including tumor cells, tumor-associated fibroblasts, and immune cells [96]. To better reconstruct this microenvironment, researchers have developed patient-derived tumor organoid models. Building on these advances, the use of induced pluripotent stem cells and endothelial colony-forming cells to construct artificial vascular systems offers new opportunities for generating endothelial models that more accurately reflect patient-specific variability [97,98]. Although some studies have incorporated immune cells, such as leukocytes, into microfluidic systems, research specifically focusing on NETs remains very limited [75].

Another important design consideration is the choice of biological samples in the perfusion system, including the choice between plasma and whole blood and the choice between samples from cancer patients and samples from healthy donors. The main advantage of plasma-based microfluidic models is their relative simplicity and high experimental controllability, making them well suited for focused studies of CAT mechanisms. However, they cannot capture key cellular events in CAT, including platelet adhesion and aggregation, leukocyte recruitment, NETosis, and cell-induced endothelial dysfunction that affects thrombus formation and stability under flow conditions [9,99]. Whole blood–based microfluidic models can capture cellular interactions involved in CAT formation under flow conditions. However, this type of models are more sensitive to preanalytical conditions: for example, anticoagulant selection, the time from blood collection to application in the chip, recalcification methods, and individual patient variability [[100], [101], [102]]. These factors can affect experimental outcomes, making standardization more challenging. Regarding sample sources, the biggest advantage of patient-derived plasma or whole blood (vs plasma or blood from healthy donors) is their high clinical relevance, because the blood of cancer patients may retain actual changes related to CAT, such as platelet hyperresponsiveness after tumor education, circulating TF-positive EVs, elevated inflammatory factors, and overall hypercoagulable state [4]. However, the limitations of patient samples are that different cancer types, stages, metastatic burdens, chemotherapy/immunotherapy, catheters, infections, and anticoagulant exposures can significantly affect the results [103,104]. In addition, the clinical sample size is usually limited, which is not conducive to the development and calibration of high-throughput platforms. In contrast, plasma or whole blood from healthy donors are better suited for mechanism research and platform standardization in the early stages of chip development. They have lower background and better reproducibility. Therefore, we propose a combined strategy. Healthy donor plasma or whole blood can be used for initial standardization of chip design, endothelialization, surface materials, flow conditions, and readouts, whereas cancer patient–derived plasma or whole blood can subsequently be used to validate whether the platform detects clinically relevant hypercoagulable phenotypes [77].

In addition, the fabrication approaches used in current CAT chips remain relatively limited and have yet to fully integrate advanced manufacturing technologies, such as 3D printing and bottom-up strategies such as self-assembly to better recapitulate the complex microenvironment. For example, in studies of cancer-associated venous thrombosis, chip models incorporating venous valve structures could be designed [105] or systems capable of spontaneously forming vascular networks could be used [106]. Moreover, current approaches are mainly used to establish microscale vascular platforms. Currently, there is a lack of large diameter vessel-on-a-chip platforms that can better mimic distant large veins associated with VTE, such as the femoral vein. Furthermore, by combining magnetic resonance imaging with 3D printing, it is possible to reconstruct patient-specific vascular geometries at sites of thrombosis, enabling precise replication of individualized vascular models [107].

Material selection also remains a major challenge. At present, most studies still rely on natural hydrogels as the primary matrix material for microfluidic chips. However, natural hydrogels often suffer from low mechanical strength and poor stability [86], which limits their use in long-term dynamic culture. In recent years, the development of hybrid hydrogels has offered a feasible solution to these limitations, as they not only enhance the mechanical properties of the material but also improve system stability while maintaining good biocompatibility [108]. Moreover, the operational lifespan of cancer-associated organ-on-a-chip platforms remains relatively limited. A thrombosis-on-a-chip system capable of stable operation for up to 1 month has already been reported [109], providing a new technical foundation for integration with tumor-on-a-chip models, thereby offering a more physiologically relevant platform for studying disease mechanisms and screening anticoagulant therapies.

Finally, key assessments of the reproducibility of CAT microfluidic platforms remain limited, and standardization between platforms has not yet been achieved. Differences in chip fabrication, cell origin, matrix materials, flow conditions, blood processing, and analytical readings can significantly affect thrombosis results, thus limiting direct comparisons between different studies. Therefore, improving the standardization of device design, operating procedures, and reporting standards is crucial for improving reproducibility and support broader application in CAT research [73,110].

## 5 Conclusions and Future Perspectives

This review summarizes existing cancer-associated organ-on-a-chip platforms and discusses their potential applications in tumor biology research, disease diagnostics, and therapeutic development. Current organ-on-a-chip studies of CAT focus on tumor-derived EVs and procoagulant factors, platelet interactions, and endothelial injury. These platforms are also used to model CAT for testing anticoagulant therapies and for predicting thrombotic risk in cancer patients. Compared with traditional preclinical models, CAT-on-a-chip platforms overcome the limitations of 2D cultures and animal models in simulating dynamic thrombus formation related to human physiology, making it possible to reconstruct the thrombotic microenvironment of cancer patients *in vitro*. Commercially available chips can improve the reproducibility and comparability of models and support large-scale drug screening. Meanwhile, custom-designed chips have the potential to evolve into standardized platforms for drug screening and risk assessment tailored to personalized treatment.

In the future, CAT-on-a-chip platforms are expected to play an important role in personalized and precision medicine. This could be accomplished by incorporating tumor tissue samples from individual patients in a chip and constructing personalized vascular systems using induced pluripotent stem cells or endothelial colony-forming cells–derived endothelial cells, combined with perfusion of the blood or plasma from the patient, to reconstruct a patient-specific thrombotic microenvironment as accurately as possible. This strategy not only helps elucidate individual mechanisms underlying CAT but also provides an experimental foundation for developing personalized anticoagulant therapies.

In terms of analytical outputs, CAT chips can be integrated with advanced multiparametric sensing technologies or artificial intelligence, enabling real-time monitoring of tumor cell behaviors and thrombus formation [111]. For example, these platforms can dynamically capture biological processes such as cell proliferation, migration, invasion, and apoptosis, while simultaneously measuring key physical parameters of thrombosis, including blood flow velocity, thrombus growth and resolution, and mechanical properties such as stiffness and elasticity [112]. Such high spatiotemporal-resolution detection provides a powerful tool for systematically understanding the interactions between tumors and thrombosis.
